# New genes of *Xanthomonas citri *subsp. *citri *involved in pathogenesis and adaptation revealed by a transposon-based mutant library

**DOI:** 10.1186/1471-2180-9-12

**Published:** 2009-01-16

**Authors:** Marcelo L Laia, Leandro M Moreira, Juliana Dezajacomo, Joice B Brigati, Cristiano B Ferreira, Maria IT Ferro, Ana CR Silva, Jesus A Ferro, Julio CF Oliveira

**Affiliations:** 1 Universidade Estadual Paulista, UNESP, Campus de Jaboticabal, Departamento de Tecnologia, 14884-900, Jaboticabal, SP, Brazil; 2 Departamento de Bioquímica, Instituto de Química, Universidade de São Paulo, USP, Av. Lineu Prestes 748, Cx. Postal 05509-900, São Paulo, SP, Brazil; 3 Alellyx Applied Genomics, Rua James Clerk Maxwell 320, 13069-380, Campinas, SP, Brazil; 4 Universidade Federal de São Paulo, UNIFESP, Departamento de Ciências Biológicas, 09972-270, Diadema, SP, Brazil

## Abstract

**Background:**

Citrus canker is a disease caused by the phytopathogens *Xanthomonas citri *subsp. *citri*, *Xanthomonas fuscans *subsp. *aurantifolli *and *Xanthomonas alfalfae *subsp. *citrumelonis*. The first of the three species, which causes citrus bacterial canker type A, is the most widely spread and severe, attacking all citrus species. In Brazil, this species is the most important, being found in practically all areas where citrus canker has been detected. Like most phytobacterioses, there is no efficient way to control citrus canker. Considering the importance of the disease worldwide, investigation is needed to accurately detect which genes are related to the pathogen-host adaptation process and which are associated with pathogenesis.

**Results:**

Through transposon insertion mutagenesis, 10,000 mutants of *Xanthomonas citri *subsp. *citri *strain 306 (Xcc) were obtained, and 3,300 were inoculated in Rangpur lime (*Citrus limonia*) leaves. Their ability to cause citrus canker was analyzed every 3 days until 21 days after inoculation; a set of 44 mutants showed altered virulence, with 8 presenting a complete loss of causing citrus canker symptoms. Sequencing of the insertion site in all 44 mutants revealed that 35 different ORFs were hit, since some ORFs were hit in more than one mutant, with mutants for the same ORF presenting the same phenotype. An analysis of these ORFs showed that some encoded genes were previously known as related to pathogenicity in phytobacteria and, more interestingly, revealed new genes never implicated with *Xanthomonas *pathogenicity before, including hypothetical ORFs. Among the 8 mutants with no canker symptoms are the *hrpB4 *and *hrpX *genes, two genes that belong to type III secretion system (TTSS), two hypothetical ORFS and, surprisingly, the *htrA *gene, a gene reported as involved with the virulence process in animal-pathogenic bacteria but not described as involved in phytobacteria virulence. Nucleic acid hybridization using labeled cDNA probes showed that some of the mutated genes are differentially expressed when the bacterium is grown in citrus leaves. Finally, comparative genomic analysis revealed that 5 mutated ORFs are in new putative pathogenicity islands.

**Conclusion:**

The identification of these new genes related with Xcc infection and virulence is a great step towards the understanding of plant-pathogen interactions and could allow the development of strategies to control citrus canker.

## Background

Citrus canker is a disease caused by the phytopathogens *Xanthomonas citri *subsp. *citri*, *X. fuscans *subsp. *aurantifolli *and *X. alfalfae *subsp. *citrumelonis *[1]. Among the three phytopathogens, the Asiatic form (*X. citri *subsp. *citri*), which causes citrus bacterial canker type A, is the most widely spread and severe, attacking all citrus varieties [2]. In Brazil, form A is the most important, being found in practically all areas where citrus canker has been detected [3]. Similarly to most phytobacterioses, there is no efficient way to control citrus canker. The only way to eliminate the disease is through the eradication of sick plants, a procedure that brings significant economical losses. By law, in São Paulo State, the main citrus production area in Brazil, it is mandated to eliminate all plants around the focus of infection in a 30 m radius if the contaminated plants are less than 0.5% of the planting field and all plants in the planted field if the contaminated plants are more than 0.5%. In the latter case, cultivation is then prohibited in the area for the next 3 years and there is no payment for lost production to the growers.

Considering the importance of the disease worldwide, especially for Brazil, a Brazilian group sequenced and annotated the complete genome of *X. citri *subsp. *citri *(Xcc) strain 306 [4], which causes citrus canker, and compared it with *X. campestris *pv. *campestris *strain ATCC 33913, the etiological agent of crucifer black rot. The citrus subspecies has 4,313 open reading frames (ORFs), of which 62.83% have been assigned function. In addition, Xcc also has two plasmids that have 115 genes, and for 55 (47.82%) of them, no role has been proposed.

Although the genome of Xcc has been characterized and annotated, the inferences made based on *in silico *analyses require experimental investigation to accurately detect which genes are related to the pathogen-host adaptation process, and which are associated with pathogenesis itself. Therefore, functional genomics studies are necessary to elucidate the machinery required for pathogen installation and proliferation in plants, and the induction of citrus canker symptoms in the host. From the functional genomic perspective, large scale analysis of mutants by inoculation in host plants allows identification of the genes required for adaptation, pathogenesis and virulence, providing a best understanding of the colonization and infection potential of the bacteria.

In this work, using transposon insertion mutagenesis [5], a library containing 10,000 mutants of the citrus canker etiological agent *X. citri *subsp. *citri *strain 306 was prepared and 3,300 mutants were analyzed after individual inoculation of host plants. Eight mutants with absent pathogenicity and 36 mutants with reduced symptoms *in planta*, at varying intensities, were identified. Mutated genes were identified by sequencing the total DNA of the mutants with altered virulence, allowing the identification of the site of insertion of the transposon used for mutagenesis.

A random selection of these genes was immobilized on a nylon membrane array and expression profiles were analyzed *in vivo *through nucleic acid hybridization to labeled cDNA probes, using targets corresponding to wild Xcc strains multiplied in non-infective (Xcc multiplied in rich culture medium) or infective conditions (Xcc multiplied in a host plant). Finally, a comparative genomic analysis of each mutated ORF region from Xcc with other sequenced *Xanthomonas *genomes allowed the identification of five interesting genomic regions, with two being exclusive to Xcc. The unique characteristics presented by these five regions suggest that they are probably new pathogenicity islands [6] in Xcc.

The implications of the proteins encoded by these mutated ORFs in host adaptation and colonization processes and citrus canker symptoms induction are discussed.

## Results

### Mutant analysis and *in vivo *pathogenicity test

To identify and characterize genes involved in pathogenicity and virulence in *X. citri *subsp. *citri *isolate 306, a library of mutants was built through random transposon insertion. To determine whether transposon insertion affected the ability of Xcc to cause disease, 3,300 mutants of this library were individually inoculated in Rangpur lime (*Citrus limonia*) plantlets. Assuming the transposon is randomly distributed along the genome in a single-copy manner, the probability of finding one transposon insertion for a certain gene can be calculated by the formula: *P *= 1 - (1 - *X*/*G*)^*n*^, where *P *is the probability of finding one transposon insert within a given gene; *X *is the length of the gene; *G *is the length of the genome; and *n *is the number of transposon inserts present in the population [7]. Based on the sequenced genome of *citri *306, and considering the main chromosome and two plasmids, the average length of each ORF in the Xcc genome is 1,019 bp [4] and the probability of finding one transposon insert for a certain gene is up to 47%. The mutants identified as having altered pathogenicity in this first round were re-inoculated and re-analyzed, resulting in a final 44 mutants showing some symptomatic variation. The mutants were grouped in five classes according to severity of the major symptoms: total absence of symptoms; watersoaking (ws); hyperplasia (hyp); necrosis (nec); and hypersensitive-like response (HR-l) [see Additional file 1].

**Figure 1:**
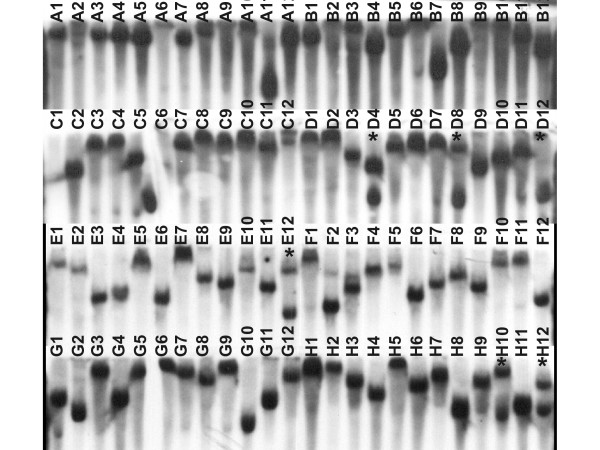
**Southern blot analysis shows transposon isertion**. X-ray film image after exposure to DNA of *Xanthomonas citri *subsp. *citri *strain 306 isolated mutant clones, previously cleaved with *Eco *RI and hybridized with the sequence of the transposon Tn5 labeled with the AlkPhos Direct RPN 3680 kit (Amersham Biosciences). Mutants with a double insert are marked with an asterisk.

The site of transposon insertion was determined by sequencing for all 44 mutants [see Additional file 1]. In 40 mutants the transposon was inserted inside an ORF and in four the insertion was at the 5'-end of the ORF, probably in the promoter region [see Additional file 1]. In addition, 5 ORFs were hit in two independent mutants (ORFs XAC0014, XAC1201, XAC1927, XAC3245 and XAC3263) and in two cases the same ORF was hit in three different mutants (ORFs XAC2047 and XAC2072), resulting in 35 different ORFs being hit. In all cases, mutants having a transposon insertion in the same ORF, irrespective of the insertion site, showed the same phenotype as determined by independent evaluations at three different times. Based on the classification proposed by the Xcc genome group http://genoma4.fcav.unesp.br/xanthomonas, the mutated genes belong to several categories: seven participate in intermediary metabolism; three are classified in the biosynthesis of small molecules; three are involved in macromolecule metabolism; two are cell structure constituents; four participate in another cellular process; two are related to mobile genetic elements; four are involved with pathogenicity, virulence, and adaptation; eight are hypothetical ORFs; and two are undefined ORFs. Therefore, among the 44 mutants there are 35 distinct mutated ORFs [see Additional file 1]. To verify that transposon insertion was random, one Southern blot analysis was evaluated. It was possible to estimate that the number of Xcc clones with double inserts in their genomes was approximately 6 in every 96 mutants of the library (Fig. 1). The same analysis was done on all 44 mutants and none of them had double inserts.

### Analysis of the growth curve *in planta *and *in vitro*

**Figure 2:**
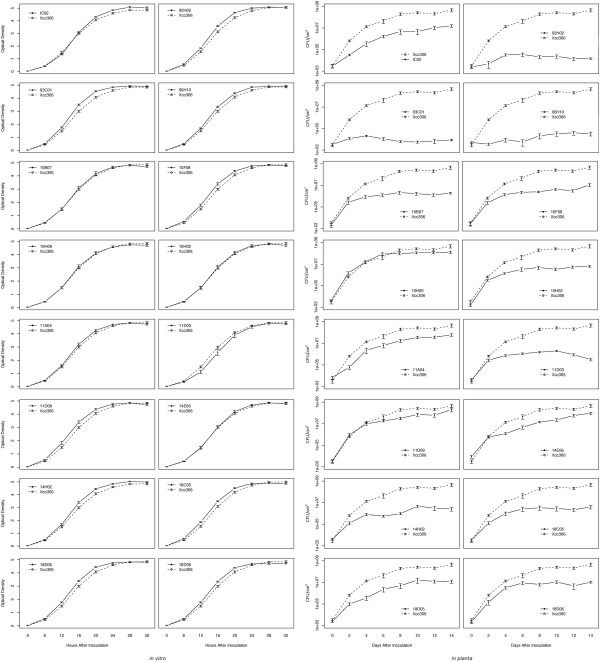
**Xcc growth curves**. Growth curves of 16 *Xanthomonas citri *subsp. *citri *mutants and wild type (Xcc strain 306) *in vitro *(left) and in citrus leaves (right).

To analyze the behavior of some mutants in terms of growth *in vitro *and *in planta*, 16 mutants were randomly selected and analyzed together with the wild type (Xcc strain 306) (Fig. 2). Although all mutants were inoculated with the same number of cells, including the wild-type strain, we observed cellular concentration differences after 2 days of growth in citrus leaves. Wild type showed cell growth until 2 days, and from that point the growth curve *in planta *remained constant at close to 10^10 ^cells/cm^2 ^of leaf area. It was possible to group the 16 mutants into five distinct patterns based on the numbers of cells per square cm: 1) mutants that showed a low concentration (10^4^–10^5^) of cells during the infection period (03C01, 02H02, 06H10); 2) mutants that showed an average concentration (10^6^–10^7^) of cells during the infection period (10B07, 10F08, 10H02, 18C05, IC02, 18D05, 18D06); 3) mutants that had high concentrations (10^7^–10^8^) of cells during the cellular infection period (10H09, 11A04, 11D09, 14E06); 4) mutants that showed a sigmoid pattern of cell concentration around 10^6 ^(14H02); and 5) mutants that had an increase in cell number equal to the wild type until the second day and then the concentration was stable (10^6^) until the 10th day, when it started to fall, reaching close to 10^5 ^on the last day (11D03). Furthermore, the mutant 18D06 also presented a sigmoid growth curve, but with a cell concentration above 10^6^.

When the same mutants were grown in culture media, it was observed that the cells grew more similarly to the wild type over time. However, among all mutants tested, the 02H02 and 03C01 mutants, which *in planta *had lower cell concentrations (probably due to the presence of some toxic metabolite or repressor of the adaptative process that affected multiplication and growth capacity), did not cause any symptoms [see Additional file 1]. Intriguingly, both genes are identified as involved with the type III secretion system (TTSS), reinforcing its importance in the disease induction process.

### Gene expression

**Figure 3:**
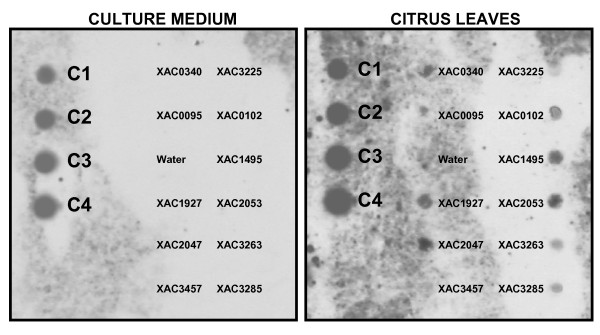
**Nucleic acid hybridization using labeled cDNA probes**. Nucleic acid hybridization using labeled cDNA probe to 11 *Xanthomonas citri *subsp. *citri *strain 306 (Xcc) genes identified as important for pathogenicity through random mutagenesis. Panel A = gene expression of ORFs when Xcc was multiplied in culture medium. Panel B = gene expression of ORFs when Xcc was multiplied in citrus leaves for 3 days. C1–C4 = controls (5 ng, 20 ng, 80 ng and 320 ng, respectively).

To show that the mutated genes are related to the altered symptoms of disease and were involved in pathogenesis (with expression dependent on plant-pathogen interaction), the expression of 11 ORFs was analyzed through nucleic acid hybridization to labeled cDNA probes in two situations: cells multiplied in culture medium (*in vitro*) and cells multiplied in citrus leaves (*in planta*) (Fig. 3).

The results indicated that the ORFs XAC0102, XAC1495, XAC2053, XAC3263, XAC3285, XAC0340, XAC0095, XAC1927, XAC2047 and XAC3225 are only expressed when Xcc is multiplied *in vivo*; it was not possible to identify expression of these ORFs when cells were multiplied *in vitro*. A single ORF, XAC3457, showed no significant expression in any of the conditions (*in vitro *and *in vivo*) (Fig. 3). The two experimental replications showed similar results.

## Discussion

Random mutagenesis through random transposon insertion in vivo in the genome has been widely and successfully used to study several microorganisms, whether pathogens or not [8-11]. Using this technique for pathogenicity and virulence studies of the causal agent of the citrus canker, a library with approximately 10,000 viable mutants of *X. citri *subsp. *citri *isolate 306 was obtained. Through this strategy, the transposon/transposase complex was inserted directly into the cells through electroporation. Southern blot analysis showed that 6.25% (6 in 96) had a double transposon insertion, which is near that expected from the description accompanying the kit used to obtain mutants, where the rate of double inserts is about 1% of the clones (Epicentre Technologies).

After individual inoculation of 3,300 mutants in Rangpur lime (*Citrus limonia*) leaves, 44 mutants were identified with some alteration in their ability to induce citrus canker symptoms. The mutated ORFs in mutants with altered pathogenicity were identified through DNA sequencing.

In this group of mutants there were genes belonging to several functional categories, including genes previously known as being involved in the pathogenesis process, such as the proteins HrpB4 and UptC and new genes XAC0340, XAC4040 and XAC2047. The symptoms caused by these mutants were also widely variable, and eight of them did not cause disease, which was confirmed by the total absence of symptoms [see Additional file 1].

In addition, to show that the mutated genes were really involved in the pathogenesis process (in other words, their expression was dependent on the plant-pathogen interaction), the expression of 11 ORFs was analyzed through nucleic acid hybridization using labeled cDNA probes in two situations: cells multiplied in culture medium (*in vitro*) and cells multiplied in citrus leaves (*in vivo*) (Fig. 3). The analyses of the blots showed that among these genes it was possible to observe the expression of most *in planta*, which denotes their importance in interaction or adaptation events during the infection process.

However, no *pthA *mutant was identified, despite Xcc having four distinct copies of *pthA*, two in each plasmid. It could be that mutation of just one *pthA *gene does not affect the establishment of Xcc in either pathogenicity or symptoms. Swarup and coworkers [12] have shown that mutation in the *pthA *gene resulted in a complete loss of virulence on citrus, but the amino acid sequence coded by *pthA *[13] is distinct from all four *pthA *copies present in Xcc 306 [4]. We used homologous recombination to disrupt each copy of Xcc *pthA *in order to determine the contribution of each copy to pathogenicity and virulence.

However, this process is not trivial, because we would first have to obtain a null *pthA *mutant, ie, a mutant with all four copies of this gene mutated. Under these conditions the adaptability of the null mutant could be tested, and, using that mutant, the contribution of each copy of *pthA *could be evaluated. Another circumstance that may have influenced the absence of identified *pthAs *mutants is the probability of having all the Xcc genes mutated in our mutant library, which was only 47%, whereas empirically, it is much easier to hit the main chromosome, due to its size, than the plasmids. So, the probability of mutating a gene in the plasmid is also very small in relation to the probability of mutating a gene on the main chromosome.

Two of the non-virulent mutants carry genes previously described as being necessary for pathogenicity, *hrpB4 *(XAC0410) and *hrpXct *(XAC1266); these two genes are part of the *hrp *(hypersensitive reaction and pathogenicity) system, which is present in most Gram-negative phytopathogenic bacteria, except for *Agrobacterium*, and is part of the TTSS [14]. Many results indirectly suggest that virulence proteins, also called virulence effectors, are injected by the pathogen directly inside the host cells through a pilus [15]. It is presumed that the effectual proteins stimulate or suppress several cellular functions of the host to benefit pathogen infection [16]. In *X. campestris *pv. *vesicatoria *(Xcv), the *hrp *cluster is 23 kb and contains six operons, *hrpA *to *hrpF *[17]. Two regulator genes, *hrpG *and *hrpX*, located outside of the larger gene cluster, are responsible for activating the expression of *hrp *genes *in planta *and in XVM2 synthetic culture media [18,19]. The mutant for *hrpB4 *in Xcv was not able to cause disease in susceptible pepper plants or the hypersensitive reaction (HR) in pepper plants carrying the respective compatible *R *gene, in the presence of *avr *in the Xcv isolate used in the study [20]. Subsequent studies confirmed that this protein, HrpB4, was not secreted; in other words, it is a protein that acts in the bacterial cell. In the same study, HrpB4 remained in the soluble protein extract while AvrBs3 was secreted by the wild isolate, which was not observed in a *hrpB4 *defective mutant.

The gene *hrpXv *(*hrpX *of *X. campestris *pv. *vesicatoria*) was characterized and its function was determined. The amino acid sequence deduced indicated similarity with proteins of the AraC family, which act in the regulation of gene expression. Mutations at position 1,335 of that gene stopped the resulting mutant from inducing disease symptoms in susceptible pepper and tomato plants and HR in resistant plants. Complementation with fragments of that gene showed that only 580 bp after the initiator codon is enough to produce a functional polypeptide. The cell concentration of *hrpX *mutants *in planta *revealed that the mutant had 10^5 ^times less bacteria than the wild type genotype [18].

These results described in previous studies of the genes *hrpB4 *and *hrpX *corroborate the results we obtained for the mutants 02H02 and 03C01, which carry mutations in the genes *hrpB4 *and *hrpXct*, respectively. These two mutants caused no disease and their growth in citrus leaves was much lower than the Xcc isolate 306 (Fig. 2). In Xcv, HrpXv acts as a transcriptional activator for genes of the group *hrp*. HrpXv is necessary for transcriptional activation of five *hrp *genes (*loci hrpB *to *hrpF*) [18]. The protein HrpB4 is necessary for the complete functionality of TTSS, since *hrpB4 *mutants are not able to secrete AvrBs3 or HrpB2 proteins in Xcv [20]. Therefore, it can be assumed that these two mutants, 02H02 and 03C01, lost their virulence because of their inability to take TTSS factors to the host cell, which are necessary for growth *in planta*, since when these mutants are reactivated in culture media, cellular multiplication is similar to that of wild type.

Another non-pathogenic mutant had mutated ORF XAC3980, which has similarity with the *Xyllela fastidiosa *gene *htrA *(high temperature requirement). First identified in *E. coli*, the locus *htrA *encodes a serine protease HtrA (also called DegP) that contains a catalytic triad (His_105_-Asp_135_-Ser_210_) required for proteolytic activity and two PDZ domains responsible for oligomerization of the protein complex, substrate recognition and substrate binding. Besides proteolytic activity, *E. coli *HtrA shows chaperone activity *in vitro *at low temperatures, where a conformational change of the protein masks the proteolytic residues. At high temperatures, the catalytic residues are accessible and the proteolytic activity of HtrA prevails. The HtrA proteases identified in *E. coli *are required for growth at 42°C and for the degradation of abnormally folded proteins in the periplasm. It was later demonstrated that HtrA degrades heat-denatured proteins, *in vivo *and *in vitro*. The very small amount of substrate for HtrA catalytic activity found *in vivo *suggests that the main biological role of the protein is the removal of nonnative, abnormally folded proteins from inside the cellular envelope. In *E. coli*, HtrA is located in the periplasm, associated with the internal membrane [21-23].

Homologous HtrA proteins are found in most bacteria, and are well conserved throughout evolution. Their impact on bacterial physiology differs among the Gram-negative bacteria. In contrast to *E. coli*, HtrA is not essential for the growth of *Salmonella enterica *serovar *Typhimurium *at high temperatures, for instance. The *htrA *mutant of *S. enterica *serovar *Typhimurium *showed reduced virulence in a murine model and reduced survival in macrophages. The phenotypic characterization of *htrA S. enterica *serovar *Typhimurium *mutant revealed a decreased tolerance to oxidative stress, which can explain the reduced survival in macrophages, where reactive intermediates of oxygen are released during the oxidative explosion. *htrA *mutants of other Gram-negative pathogenic bacteria, such as *Yersinia enterocolitica*, *Klebsiella pneumoniae *and *Brucella abortus*, are sensitive to both high temperatures and oxidative stress [21]. Moreover, *htrA *mutants of *Y. enterocolitica *and of *B. abortus *show reduced virulence in murine models.

In *Listeria monocytogenes*, transcriptional analyses in an *htrA *mutant revealed that the gene *htrA *is not induced in response to thermal shock, but rather to stress caused by low pH and penicillin G. In addition, a significant virulence decrease was detected in this mutant, revealing that HtrA is very important for the complete virulence of *L. monocytogenes *in mice. Recently, an *htrA *mutant of *L. monocytogenes *10403S was shown to be sensitive to oxidative stress and puromycin at high temperatures, and showed a reduced ability to produce biofilms and attenuated virulence in mice [24].

However, the attenuated virulence of Gram-negative htrA mutants remains unclear since they are more susceptible to stress than the isolated parent is; the mutants may also be less viable in host tissues, which will trigger several types of stress to the invading cell. Besides, it is believed that the chaperone and processing functions of HtrA protein are necessary for folding secreted proteins, or that HtrA may be involved in the oligomerization and exportation of virulence factors [22,23]. Therefore, the *htrA *gene has been shown to be essential for the complete virulence of many pathogens.

On the other hand, HtrA is not essential for bacterial growth under unstressed conditions, so it is a potential target for anti-pathogen drugs, including those that inhibit virulence rather than killing bacteria or stopping bacterial growth. It is assumed that anti-pathogen drugs reduce the pressure for development of resistance, which is an extremely important trait when it comes to agricultural pests, because such a drug must be applied over large areas and produces high selection pressure. Moreover, not killing the target makes this kind of drug type ecologically sustainable, because it cannot favor bacterial evolution [25-27]. Thus, the data in the literature and the results of the present study allow the assumption, for the first time, that the gene *htrA *(XAC3980) of Xcc is part of an important process during pathogenic colonization, being necessary for the complete virulence of the pathogen.

Heme groups are responsible for carrying out a wide variety of biological functions in prokaryotes and eukaryotes. These groups are essential for respiration, oxygen metabolism and electron transport, as well as for prosthetic groups, hemoglobulins, hydroxylases, catalases, peroxidases and cytochromes [28]. More recently, new roles for heme groups have been described as biosensors of diatomic gases [29,30] and modulators of protein activity [31]. Protoheme biosynthesis involves seven enzymatic steps, starting from the universal precursor delta-aminolevulinic acid (ALA). Other heme groups that cells need are obtained from protoheme modifications. In the step before production of protoheme, an iron ion (Fe^2+^) is inserted in protoporphyrin IX, catalyzed by ferrochelatase [32].

One of the many roles played by protoheme in the cell is the constitution of cytochrome. Type c cytochromes, which contain a covalent heme c group, are widely distributed in organisms, in which they play a role in photosynthesis and electron transport from the respiratory chain. Most type c cytochromes of *E. coli *and *S. enterica *serovar *Typhimurium *are c_552 _cytochromes, which are comprised of six covalently bound heme groups and are located in the periplasmic space where they act as dissimilatory nitrite reductase [33].

Therefore, heme groups are used in basic metabolism for energy production, in the electron transport chain in an aerobic pathway, and in the nitrite reduction complex in an anaerobic pathway. The interruption of heme group production thus presumably affects the electron transport chain, which hinders the use of oxygen or nitrate as final electron recipients by cells. If this hypothesis is true, it explains why the mutant 11D09, which carries the interrupted ORF XAC4040, a delta-aminolevulinic dehydratase (*hemB*), does not cause disease and shows total absence of symptoms.

Proteomic analysis showed that proteins involved in glycolytic and related pathways and fermentation are over-expressed in *hemB *mutant cells, which show exponential growth, compared to the parental strain [34], indicating that the mutant *hemB *produces energy only from phosphorylation at the substrate level *in vitro*. Thus, the observation that the mutant 11D09 is multiplied *in planta *(Fig. 3) is explained by the use of carbon sources for the production of anaerobic ATP, or even by the use of the hemes produced by the plant.

So, considering the information available in the literature, the *hemB *mutant can survive *in vitro *and *in planta *by producing energy from hexoses or from intermediate compounds such as pyruvate, producing lactate, acetyl-CoA, producing ethanol, or L-arginine, producing CO_2 _+ NH_4_. Moreover, its survival may also depend on the uptake of heme from the environment through specific carriers for this purpose present on Xcc, *CcmA*, *CcmB*, *CcmC *and *cycZ *(XAC2323, XAC2324, XAC1679 and XAC2325, respectively). In the first three cases ATP production is low and for the latter the possibility of spending a lot of energy to transport the heme compound into the cell, which also results in a low ATP balance. So, in all cases, the cell would probably optimize energy expenditure for survival. Whereas the process of pathogenesis demands the production of various enzymes, proteins and other compounds, these observations suggest that these processes will not be realized because of their high energy consumption. Consequently, although the cell survives reasonably well, both *in vitro *and *in planta*, it will not develop the disease and thus no external symptoms will be observed. Finally, the fact that the mutant *hemB *had a growth curve *in planta *very similar to wild type may be an indication that it is performing aerobic metabolism due to internalization of heme compounds from the host and not causing the disease because the energy balance is not favourable, since transport through the membrane consumes so much energy.

Histidine kinases are proteins that can play a major process in bacterial metabolism. These proteins, together with their cognate response regulators (RR), can be part of two component systems (TCS), which constitute a signal transduction process in which bacteria sense, respond, and adapt to changes in their environment or intracellular state. Signal transduction starts when a histidine kinase senses a signal, e.g., by binding or reacting with a signaling molecule or due to a physical stimulus, and phosphorylates downstream proteins in the phosphorylation cascade that modulate the activity of a final set of protein targets, which then modulate protein activity or differential gene expression. Based on their components, two TCS exist: prototypical and phosphorelay systems [35].

In the phosphorelay TCS pathway, a stimulus activates autophosphorylation of a hybrid histidine kinase, namely, a histidine kinase containing a phospho-accepting receiver domain, typically at the C-terminal end of the protein. The catalytic and ATPase (HATPase – PF02518 – Pfam A accession – http://pfam.sanger.ac.uk/help) domain of the histidine kinase is responsible for binding ATP and catalyzing autophosphorylation of a conserved histidine found within the dimerization and histidine phosphotransferase (HisKA – PF005121) domain. The HisKA domain mediates homodimerization and serves as the phosphodonor for a C-terminal receiver domain (response regulator – PF00072), similar to that found in response regulators. A histidine phosphotransferase (HPT – PF01627) then shuttles the phosphoryl group from the hybrid kinase to a soluble response regulator containing an output domain through protein-protein interaction or protein-DNA interactions leading to differential gene expression [36-38].

Xcc has a gene that codes for a histidine kinase, XAC3673, that is similar to the hybrid histidine kinase found in many TCS. XAC3673 has HisKA, HATPase, and response regulator domains [see Additional file 1]. An analysis using Psort [39] found that the predicted protein from XAC3673 is localized on the bacterial inner membrane and a blastp search result [40] found that the first 60 amino acids only match sequences from *X. citri *subsp. *citri*, *X. campestris *pv. *vesicatoria *and *X. oryzae *pv. *oryzae*, indicating that the N-terminal sequence is exclusive to *Xanthomonas*. The blastp result from amino acids 200 to 578 at the C-terminus found similarities with RpfC protein from Xcc, and with many RpfC proteins that are involved in quorum sensing signaling mediated by a diffusible signal molecule DSF (diffusible signaling factor). This quorum sensing mechanism plays a key role in the regulation of xanthan (EPS) biosynthesis, gene expression, motility, adaptation, and bacterial virulence [41]. RpfC from Xcc (XAC1878) has the same three domains: HisKA, HATPase, and the response regulator, as well as an Hpt domain. Furthermore, RpfC is a bacterial inner membrane protein [42].

In *Xanthomonas*, the RpfC and RpfG proteins are a two-component system implicated in DSF perception and signal transduction. At a low cell density, the DSF sensor RpfC forms a complex with the DSF synthase RpfF through its receiver domain, which prevents the enzyme from effective synthesis of the DSF signal. In this step, DSF is synthesized at basal levels. But when the cell density increases, extracellular DSF increases, too. So at a high cell density, accumulated extracellular DSF interacts with RpfC and induces a conformational change in the sensor, which undergoes autophosphorylation and facilitates release of RpfF and phosphorelay from the sensor to its response regulator RpfG. Now, RpfF, together with RpfB, can induce the production of DSF, and RpfG can induce EPS biosynthesis, gene expression, motility, adaptation, and bacterial virulence [41].

The RpfC mutants produce significantly attenuated virulence factors, but synthesize about 16-fold higher DSF signal than the wild type [42,43], whereas mutation of *rpfF *or *rpfB *abolishes DSF production and results in reduced virulence factor production [44,45]. Deletion of either *rpfC *or *rpfG *decreases the production of EPS and extracellular enzymes [42,45]. Based on these results, it was proposed that RpfC/RpfG is a signal transduction system that couples the synthesis of pathogenic factors to sensing of environmental signals that may include DSF itself [42].

Nevertheless, the current knowledge about the signal transduction pathway downstream of RpfC/RpfG is still little. Recent study presented evidence that the HD-GYP domain of RpfG is a cyclic di-GMP phosphodiesterase that degrades the second messenger bis-(3'-5')-cyclic dimeric guanosine monophosphate [46]. Furthermore, RpfG interacts with GGDEF domain-containing proteins [47]. The same authors, Andrade and coworkers, showed that RpfG interacts with sigma factor 54, NtrBC, and other regulatory proteins.

We found a mutant, 18D06, in our mutant library in which XAC3673 was knocked out; the mutation site is located inside the response regulator domain [see Additional file 1]. This mutant was observed at a high concentration *in planta *(Fig. 2) but with no symptom development [see Additional file 1]. Despite the ability of a hybrid histidine kinase to be involved in phosphorylation of any pathogenicity related gene, we believe that this protein plays a more sophisticated role in the virulence process in Xcc.

Considering the data presented above, namely a protein localized on the inner membrane with high similarity with RpfC, a *Xanthomonas *exclusive amino terminus, and high mutant cells concentration *in planta*, led us to propose this role for XAC3673 in Xcc: participation in the perception and transduction of signals in the quorum sensing system in this *Xanthomonas citri *subsp. *citri*. Besides these features, the fact that the response regulator domain (PF00072) from XAC3673 interacts with the domains CheB_methylest (PF01339), Response_reg (00072), Trans_reg_C (PF00486), GGDEF (00990), Hpt (PF01627), P2 (07194), Sigma54_activat (00158), and ANTAR (PF03861) [38] gave us more data on which to base this hypothesis.

**Figure 4:**
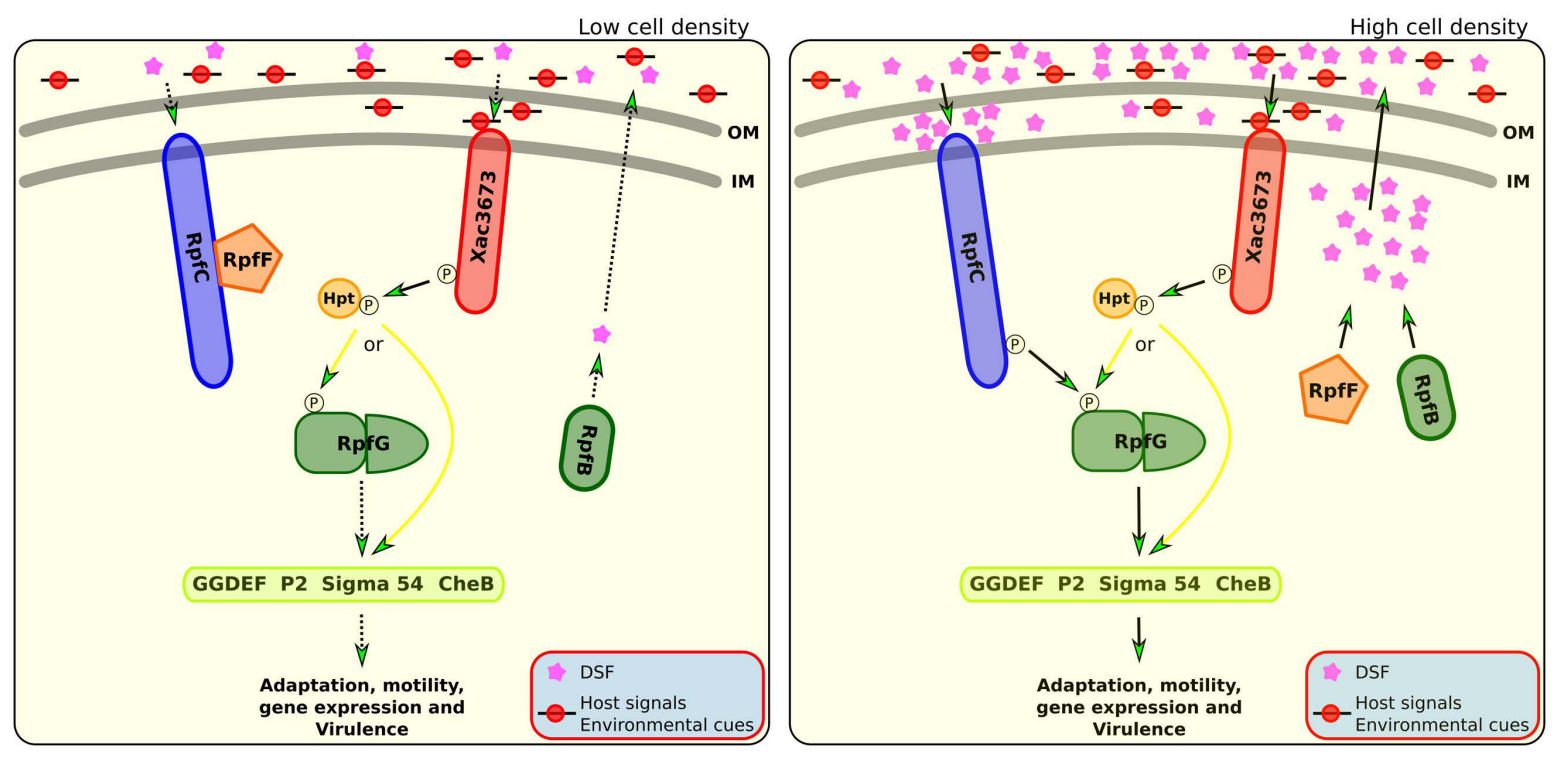
**Schematic representation of a suggested DSF signaling model including XAC3673**. Schematic representation of a suggested DSF signaling model including XAC3673. At a low cell density, the DSF sensor RpfC forms a complex with the DSF synthase RpfF, which prevents the effective synthesis of the DSF signal. At a high cell density, accumulated extracellular DSF signal interacts with RpfC, which undergoes autophosphorylation and facilitates release of RpfF and phosphorelay from the sensor to its response regulator RpfG. The event boosts DSF biosynthesis and induces the expression of the EPS and extracelular enzymes. In either, low or high cell density, there may be other stimuli (signals), in the extracellular environment from the host or the environment, regardless of the bacterial cellular concentration. The synthesis of Xcc virulence factors only start after the perception of such signals. XAC3673, through a phosphorylation cascade, relays this information to RpfG or to another protein downstream (arrows with yellow lines). A mutation in XAC3673 prevents the transduction of signals from the environment or host, and thus, the virulence factors are not produced, even in the presence of all functional *rpf *genes and with a high cell concentration. The solid arrow indicates signal flow or signal generation and the dashed arrow indicates basal signal generation or no signal flow. OM = outer membrane; IM = inner membrane.

XAC3673 protein can be on the inner membrane and the amino terminus could act as a sensor to perceive host or environmental signals. After signal reception, XAC3673 may be autophosphorylated. The HisKA domain serves as the phosphodonor for the C-terminal receiver domain (response regulator). A histidine phosphotransferase then shuttles the phosphoryl group from the hybrid kinase to a cytoplasmatic response regulator, which could be RpfG or another downstream protein in the signaling chain carrying at least one of the eight domains with which it could interact [38]. Thus, we are supposing that XAC3673 is an important required member of the signaling transduction process in Xcc (Fig. 4), acting together with RpfC/RpfG and required for complete virulence. When RpfC, RpfG or XAC3673 is not functional, virulence is abolished, but the mutant is viable. Another observation that we think is important is the site of the mutation on XAC3673: the response regulator domain. The response regulator domain in RpfC and XAC3673 are very similar, indicating that they could share the same protein-protein interactions with RpfG or with other proteins in the downstream signaling pathway. Figure 4 summarizes our hypothesis about the proposed role of XAC3673 in quorum sensing in Xcc.

**Figure 5:**
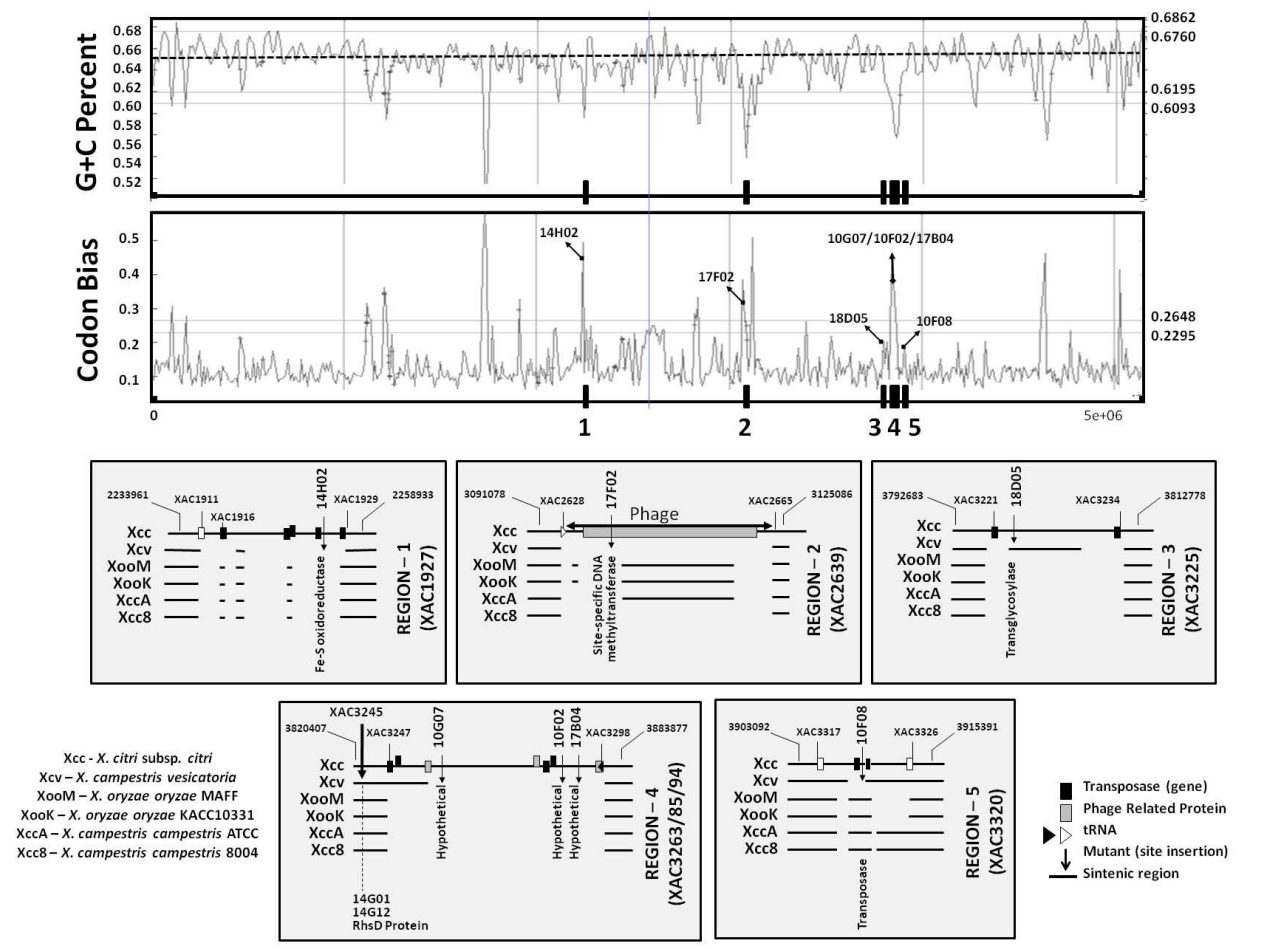
**Xcc genome exclusive regions**. Determination of possible Xcc exclusive regions on the basis of analysis of mutant (upstream and downstream) flanking regions. Five regions were found (1–5), three very close to each other (3–5). Notice that all regions have a GC content and codon bias sequence that differs from the rest of the genome profile. Details of each region are shown. Note that regions 1 and 4 are Xcc exclusive regions.

Finally, we compared the Xcc genomic regions in which the mutated ORFs are located to other bacterial genomes. Basically, we used the sequence analysis tool BLAST [40] to compare these Xcc regions with the corresponding regions of the genomes of five other *Xanthomonas *species: *X. campestris *pv. *vesicatoria*, *X. oryzae *pv. *oryzae *MAFF, *X. oryzae *pv. *oryzae *KACC10331, *X. campestris *pv. *campestris *ATCC 33913 and *X. campestris *pv. *campestris *8004. At the end of this comparative analysis, five regions were highlighted (Fig. 5). Region 1 (delimited by ORFs XAC1911 and XAC1929) and region 4 (delimited by ORFs XAC3260 and XAC3298), which hold respective knockout ORFs XAC1927, and XAC3263, XAC3285 and XAC3294, are exclusive to Xcc. However, regions 2, 3 and 5, which contain respective knockout ORFs XAC2639, XAC3225 and XAC3320, are present in at least one of the other studied genomes, but not in all (Fig. 5). In addition, some characteristics of these regions, such as abnormal variation in nucleotide composition (GC percent, dinucleotides, codon usage) and the appearance of relaxases, mobilization proteins, phages, transposons and integrases (Fig. 5), are good indicators of viable lateral transfer regions [48]. Indeed, recently Lima and coworkers [49], when examining the Xcc genome in search of viable Xcc genomic region candidates for lateral transfer regions, also concluded that regions 2 and 5 (regions 20 and 23 respectively [49]) are genomic islands, which supports the hypothesis. The other three regions, 1, 3 and 4 (Fig. 5), have no corresponding sequences or regions in the work of these authors, but regions 3 and 4 are very similar to the XAUC12 and XAUC13 regions identified by Moreira and coworkers [50].

Being exclusive to Xcc, regions 1 and 4 deserve special attention (Fig. 5). The XAC3263, XAC3285 and XAC3294 ORFs, which encode hypothetical proteins of unknown roles and that showed different expression patterns under the conditions mentioned above, are in region 4. This region is found in the ORF XAC3260 (plasmid mobilization protein) and extends until XAC3298 (one integrase downstream of a tRNA_*Gly*_), totaling 37.546 kb. In terms of composition, this region has mainly hypothetical ORFs. The encoded product of one of these ORFs (XAC3266) interacts with the protein VirD4, a gene classically correlated with the type IV secretion system [51]. It is important to emphasize that upstream of this region there are ORFs that encode a virulence regulator (xrvA) (XAC3256), transposases (XAC3247) and regulated component colSR (XAC3249/50). Most curious is the ORF XAC3245, which encodes an rhsD protein, and the respective mutants also show massive reduction of the necrosis phenotype (mutants 14G01 and 14G12), which also was upstream of region 4 (Fig. 5). In addition, for ORFs XAC3263, XAC3285 and XAC3294, no classically described domain was found in the probable proteins encoded by these hypothetical ORFs and an analysis by Psort [39] revealed that they are cytoplasmic proteins and, in a similar manner, no clusters of orthologous groups (COGs) of proteins [52] were found, demonstrating that there is no similarity with any other sequences.

In a different way, region 1 also calls attention by containing 5 transposases, alternating with hypothetical ORFs (Fig. 5). Among ORFs with functions previously predicted by genome annotations, there is ORF XAC1927, which encodes an Fe-S oxidoreductase that has been knocked out, and another that encodes a hemolysin related protein (XAC1918). For this ORF, XAC1918, it has also been proven experimentally that its product is connected to the *virD4 *product [51]. Related to the structural aspect, this region, besides having abnormal variations in the constitution of its nucleotides, is located between two major conserved gene clusters related to flagellum biosynthesis and regulation. In other organisms, including some *Xanthomonas*, these genes are concatenated, evidence that reinforces the hypothesis that this region was acquired by a lateral transfer process.

Because of all of these peculiarities, these five regions qualify as strong candidates for classification as probable lateral transfer islands and, in this particular case, as probable pathogenicity islands, as they present many of the typical characteristics found in these regions [6].

Finally, another three ORFs were analyzed under this profile (data not shown): ORF XAC2639, which encodes a site-specific DNA methyltransferase, is inserted in a region that is characterized as a phage insertion region; the sequence is partially found only in the two sequenced species of *X. oryzae *and in *X. campestris *ATCC 33913; ORF XAC3225, which is in a region only found in *X. vesicatoria*; and ORF XAC3320, which encodes one transposase only absent in the *X. vesicatoria *strain.

In short, three of the seven ORFs described as candidate genes to be present in lateral transfer islands were analyzed in terms of expression levels and conditions. It was observed that they play important roles in plant-pathogen interrelations, because they are only expressed when cells are multiplied *in planta*. The culture medium does not contain compounds present in plants, and for this reason, it did not induce expression. However, the observation that mutants for these genes showed reduced virulence and symptom alterations supports their importance in the interaction with the host.

These results corroborate the altered pathogenicity of the mutants studied here when inoculated in a host plant, indicating that the products of these genes are important for pathogen establishment and development in the host.

## Conclusion

The experiments described in the present study represent the first attempt to use a high-throughput mutagenesis analysis method to identify a wealth of genes that contribute to Xcc virulence. These results allowed identification of new putative virulence factors, as well as novel potential targets for drugs in this strain, especially the genes present in the Xcc exclusive putative pathogenicity island.

## Methods

### Bacterial strains, culture media and growth conditions

Xcc strain 306 [4] was maintained in phosphate buffer at room temperature during all experiments. Growth experiments were performed in either TSA medium (10 g/L tryptone, 10 g/L sucrose, 1 g/L sodium glutamate) or NB medium (3 g/L beef extract, 5 g/L peptone) at 28°C, with addition of agar (15 g/L) where solid medium was required. Cells were grown in test tubes containing 3 mL of culture medium, at 28°C with shaking at 200 rpm, or in Petri dishes in an incubator at 28°C. When required, kanamycin or ampicillin was added to the culture medium to a final concentration of 100 *μ*g/mL. *E. coli *strain DH10B was maintained at -80°C on Luria-Bertani (LB) medium containing 12.5% (v/v) glycerol and was grown on LB medium at 37°C with shaking at 200 rpm.

### *In vitro *mutagenesis

A set of Xcc strain 306 mutants was obtained by random insertion of the Tn5 transposon. The transposon was inserted by electroporation (2500 V, 25 *μ*F, 200 ohms, 0.2 cm cuvette width) with an EZ::Tn5 KAN-2 Tnp Transposome Kit, according to the instructions of the manufacturer (Epicentre Technologies). Transformed colonies were selected on TSA culture medium containing kanamycin (transposon selection marker) and mutants were picked and transferred individually to 96-well microtitre plates containing TSA culture medium with kanamycin and 20% (v/v) glycerol. After growing for 2 days at 28°C with shaking at 200 rpm, the plates were stored at -80°C.

### *In vivo *virulence test

Mutants were individually multiplied in solid TSA medium with kanamycin under the conditions previously described. In each 14-cm Petri dish containing solid culture medium, it was possible to multiply 96 mutants by using a 96-pin replicator. After growth for 72 h, each mutant was individually collected from the plate and placed into 1.5 mL polypropylene tube. The cellular concentration was adjusted by the addition of double-distilled water to an optical density of 0.3 at 600 nm, which is equivalent to approximately 10^8 ^CFU/mL. The bacterial suspension was then infiltrated using a syringe to two points of the left abaxial side of young Rangpur lime leaves, which were used as host for the *in vivo *pathogenicity tests. The wild-type strain, used as a positive control, was inoculated on the right side of the same leaf using the same concentration and conditions. After inoculation, plants were grown in a chamber at 28°C with artificial light. The development of citrus canker symptoms in host plants was evaluated every day, from the 3^rd ^to the 21^st ^day after inoculation. Mutants that showed different symptoms or levels of virulence from the wild-type strain were selected in this first screening. Each mutant selected was re-inoculated three times to confirm the results. All the symptoms were registered by digital photographs, including the ones presented by the wild-type strain.

### Total DNA extraction from *Xanthomonas citri *subsp. *citri*

Mutant clones were multiplied in 96-well microtitre plates containing 1 mL of TSA culture medium and kanamycin for 48 h at 28°C and 200 rpm. Plates were then centrifuged for 30 min at 3,000 *g *at room temperature. The supernatant was discarded and 500 *μ*L of freshly prepared washing buffer (10.0 mM Tris-HCl pH 8.8, 3.0 mM KCl, 1.25 mM NaCl) was added to the cell pellet of each well. The cell pellet was resuspended by strong vortex agitation and centrifuged at 3,000 *g *for 15 min at room temperature. The washing step was repeated and the pellet was then resuspended by strong vortex agitation in 500 *μ*L of buffer D (25 mM sodium citrate, pH 7.0, 5.0 g/L Sarcosyl, 4 M guanidine isothiocyanate) and kept in a water bath at 65°C for 1 h. After cell lysis, 210 *μ*L of buffer P (667 mM Tris-HCl (pH 7.5), 833 mM NaCl, 83 mM EDTA (pH 8.0)) was added to each well and the plates were agitated and centrifuged at 3,000 *g *for 30 min at room temperature. A 550-*μ*L aliquot of the supernatant was transferred to new 96-well microtitre plates and centrifuged at 3,000 *g *for 15 min at room temperature. After this procedure, 150 *μ*L of the supernatant was carefully transferred to a 96-well ELISA plate, avoiding transfer of pellet debris. To isolate DNA from the solution, 130 *μ*L of cold isopropanol (-20°C) was added to each sample, which was then kept at -20°C for 12 h. The plates were centrifuged at 3,000 *g *for 45 min at 4°C and washed twice with 200 *μ*L of 70% ethanol, with the plates being centrifuged at 3,000 *g *for 20 min at 4°C after each wash. The pellets were dried, resuspended in 40 *μ*L of TE buffer (10 mM Tris-HCl, pH 8.0, 0.1 mM EDTA) containing 1 *μ*g/mL of RNase A and kept for 1 h at 37°C. DNA quality and concentration were evaluated in a 0.8% agarose gel by comparing experimental samples with a known concentration of a high-quality DNA sample.

### Identification of mutated genes

#### DNA cleavage and fragment cloning

Total DNA from each Xcc mutant and from plasmid vector *p*BlueScript II SK DNA (Stratagene) was cleaved in a total volume of 25 *μ*L with *Eco *RI, *Sac *I or *Sac *II, as recommended by the enzyme manufacturer (New England Biolabs). These enzymes do not cut inside the transposon sequence and were used in pairs. After cleavage, the restriction enzymes were thermally inactivated and the fragments were cloned into the vector cleaved with the same enzyme pair combinations in a 500-*μ*L microcentrifuge tube containing 3.5 *μ*L of sterile double-distilled water, 1 *μ*L of 10× enzyme buffer, 0.5 *μ*L (200 U) of T4 DNA ligase, 2.0 *μ*L (15 *μ*g) of total mutant DNA cleavage product and 3.0 *μ*L (5 *μ*g) of the vector cleavage reaction product. The ligation reaction was carried out at 16°C for 12 h and used to transform electrocompetent *Escherichia coli *DH10B cells [53]. This strategy yields clones containing the transposon flanked by the mutated gene.

#### Transformation of *Escherichia coli *with the recombinant plasmid

An aliquot of the ligation reaction (2 *μ*L) was added to 40 *μ*L of *E. coli *DH10B electrocompetent cells and electroporated as described before. Subsequently, the electroporated *E. coli *cells were transferred to a 15 mL screwcap polypropylene tube and 1 mL of SOC culture medium (20 g/L tryptone, 5 g/L yeast extract, 10 mM NaCl, 25 mM KCl, 10 mM MgCl_2_, 10 mM MgSO_4_, 20 mM glucose) was added to the tube. The cells were constantly shaken (200 rpm) at 37°C for 1 h. A 200-*μ*L aliquot was inoculated in a Petri dish containing LB culture medium with kanamycin, 100 mM IPTG and 40 mg/mL X-Gal [53]. After growth in an incubator for 12 h at 37°C, three individual colonies of each mutant were picked and transferred to 96-well microtitre plates containing LB culture medium with kanamycin and grown for 12–14 h at 37°C. Plasmids were extracted by an alkaline lysis method [53].

#### Sequencing of mutated genes

The extracted plasmid DNA was sequenced using BigDye terminator v3.0 (Applied Biosystems). To map the transposon insertion in each mutant, two independent sequencing reactions were performed, each using one of the oligonucleotides KAN-2 FP-1 or KAN-2 RP-1 (Epicentre Technologies). With this procedure, genome regions flanking the transposon were sequenced. The resulting sequences were analyzed by bioinformatics to remove possible transposon sequence, and aligned with the genome of *X. citri *subsp. *citri *isolate 306 to identify the mutated gene. Sequences were aligned through the algorithm BLASTn [40].

### Southern blotting

Southern blotting was used to verify the rate of double insertions and randomness of transposition in the Xcc genome in our mutant library. A plate containing 96 mutants was randomly selected from the mutant library and total DNA was extracted as described above. DNA samples were cleaved with the restriction enzyme *Eco *RI and separated in a 1% agarose gel in TBE buffer for 12 h at 35 V. At the end of this process, the gel was stained with ethidium bromide and the image was documented. The DNA was transferred from the gel to a Hybond N^+ ^nylon membrane, following the manufacturer's instructions (Amersham Biosciences). Transposon Tn5 DNA (100 ng) was labeled using an AlkPhos Direct RPN 3680 labeling kit and probe signals were detected with a Gene Images CDP-*Star *RPN 3510 kit (Amersham Biosciences), according to the manufacturer's instructions. The membrane was finally exposed to X-ray film, stored at room temperature for 1 h and developed using the GBX kit (Kodak). The film was analyzed under a white light transilluminator. Two independent hybridizations were carried out to confirm results. The same mutants were independently multiplied and the process was fully repeated.

### Determination of *Xanthomonas citri *subsp. *citri *growth curves *in planta*

Eight mutants with altered virulence (02H02, 03C01, 06H10, 11D09, 18C05, 18D06, 11D03, 10H02) and a wild-type strain (isolate 306) were chosen for determination of growth curves *in planta*. These mutants carry knock-out versions of ORFs XAC0410, XAC1266, XAC0789, XAC4040, XAC0340, XAC3673, XAC1201 and XAC0095, respectively, created by transposon insertion. Mutant and wild-type strains were multiplied in TSA culture medium as above described. After growth, an aliquot of each was transferred to 1.5 mL microcentrifuge tubes containing 1 mL of sterile distilled water. After complete dissolution of the cell pellet, the concentration was adjusted to an OD of 0.1 at 600 nm then diluted to OD 0.01 (approximately 10^4 ^CFU/mL). Using a syringe, an orange leaf was infiltrated with each bacterial suspension. Quantitative analyses were performed 0, 2, 4, 6, 8 and 10 days after inoculation. The number of cells per leaf area was measured in three disks of 1 cm^2 ^from each inoculated leaf. With a pestle, leaf disks were ground in 1 mL of double-distilled sterile water. Serial dilutions of 10^-1 ^to 10^-7 ^were prepared and 10 *μ*L of each dilution was used to inoculate TSA culture medium containing kanamycin (except for the wild type) using a microculture technique [54]. Plates were kept at 28°C for 2 days, and isolated colonies (cells) were counted. The experiment was repeated independently three times.

### Gene expression analysis detected through nucleic acid hybridization using cDNA probes

Bacterial cells were grown in a plate for 72 h under the above conditions. To obtain RNA from cells growing in the culture media, suspension of Xcc 306 cells was adjusted for OD 0.3 at 600 nm, and 1 mL was inoculated in 50 mL liquid NA medium, then inoculated for 48 h in a shaker (200 rpm) at 28°C. Cells were transferred to 30 mL tubes and total RNA was extracted with TRIzol reagent (Invitrogen) as recommended by the manufacturer. Samples were treated with DNase I (Invitrogen) according to the manufacturer's instructions, and then stored at -80°C until use. To obtain RNA from cells growing in the host, at least 20 citrus leaves were infiltrated with a suspension of Xcc 306 cells (OD 0.3, 600 nm). At 3 days after inoculation, leaves were collected and minced in cold distilled water, in order to facilitate the exudation of bacterial cells to the liquid medium. After 10 min of agitation in an ice bath, the cut leaves were removed and bacterial cells were collected in a Corex tube by centrifuging at 5,000 × *g *for 10 min. Total RNA extraction and DNase I treatment were perfomed as described above. Eleven primer pairs (Table 1) were designed for the amplification of the 11 Xcc ORFs for which some sort of virulence deficiency was detected after mutation. The amplification products were used in a nucleic acid hybridization using labeled cDNA probe technique as described below in order to assess possible differential gene expression in these mutants.

**Table 1:** Primers used in nucleic acid hybridization. Primers and respective *Xanthomonas citri *subsp. *citri *ORFs employed in the amplification of ORFs used in nucleic acid hybridization using labeled cDNA probes.

ID	ORF	Size (bp)	Forward Primer	Reverse Primer
1	XAC0340	432	gATACCCCATATgAATgCgAT	CAgCgCCAAgCTTATgCCATg
2	XAC0095	222	AggAgAgCCATATgCACgACg	TTgCATCgAATTCAgTgCgTT
3	Water			
4	XAC1927	1.179	ggAgTCTCATATgCTgACgCg	CCggTACCTCgAgTgTCATg
5	XAC2047	1.224	ggATgggCATATggCAAgCAg	AACggAgAATTCATgCCTgCg
6	XAC3457	648	CggCATTCATATgACTCCCTT	CATCTgCggATCCACATTACT
7	XAC3225	1.278	TCgggTgTCATATgATCATgC	ATgCAgCCTCgAgCgTACATC
8	XAC0102	660	ATCAgCTgCggCAACAggTg	AgCgggTCAgTCTgAAgACACg
9	XAC1495	405	ATATCCTCATATgTCCAAATC	ATTTgACTCgAgACggATCAg
10	XAC2053	2.361	gTggTgCCTTACggTTTCAg	CAgATCAgCCCATTACgACg
11	XAC3263	537	AACCACATCgCTTTCTTCCC	TggATCgTTTgCTgACgg
12	XAC3285	429	ATggACTTCATgCACgACC	gAACTggAAACCTggATgAgC

Xcc 306 DNA samples were used in PCR performed using an initial denaturing step of 94°C for 3 min, followed by 35 cycles comprising a denaturing step of 94°C for 30 s, an annealing step at 48°C for 30 s, and a polymerization step at 72°C for 2 min. A final polymerization step of 72°C for 4 min was run, and then samples were kept at 4°C until use. The amplification reaction was carried out with 0.2 *μ*L of DNA, 5 *μ*L of 10× buffer, 1.0 *μ*L of 50 mM MgCl_2_, 1.0 *μ*L of 10 mM dNTP, 2.5 *μ*L of each primer, 37.5 *μ*L of sterile double-distilled water and 0.3 *μ*L of *Taq *DNA polymerase (Invitrogen). An aliquot (5 *μ*L) of the amplification product was electrophoresed in a 1% agarose gel, stained with ethidium bromide and visualized using an ultraviolet light transilluminator. The reaction was considered positive for a gene when the obtained product's size was as expected.

An aliquot of 400 ng of the amplified PCR product was denatured by addition of one volume of 0.4 N NaOH followed by heating at 70°C for 10 min. The solution was put into an ice bath for 5 min and an equal volume of cold 2 M ammonium acetate (pH 7.0) was added. Meanwhile, positively charged nylon membranes, previously equilibrated in 6× SSC (0.9 M NaCl, 90 mM sodium citrate) for 30 min, were mounted in a Bio-Dot apparatus (Bio-Rad). To assure denaturation of DNA, 500 *μ*L of 0.4 N NaOH was applied under vacuum to each well of the transfer apparatus. Denatured DNA samples representing ORFs of interest were then transferred under vacuum to the membrane. Samples were quickly washed in 2× SSC and the DNA was fixed with an ultraviolet crosslinker (Ultraviolet Crosslinker Model CL-1000, UVP), according to the membrane manufacturer's recommendations (Amersham Biosciences). The membrane was placed in a plastic bag, sealed and kept in a refrigerator until use.

Approximately 5 *μ*g of *X. citri *subsp. *citri *(isolate 306) total RNA, obtained from cells grown in culture medium or *in planta *and treated with DNase I, were used individually for the synthesis of first-strand cDNA with the SuperScript First-Strand synthesis system for RT-PCR (Invitrogen) according to the manufacturer's instructions. After synthesis of first-strand cDNA, 2 U of RNase H was added to each sample. Samples were gently shaken, kept at 37°C for 20 min and then stored at -20°C until use. The first-strand cDNA of each sample was labeled with alkaline phosphatase using the AlkPhos Direct Labeling kit (Amersham Biosciences). The membrane was pre-hybridized, hybridized and submitted to post-hybridization washes using the same kit, following the manufacturer's instructions. Detection was performed with CDP-*Star *(Amersham Biosciences) for 5 min at room temperature. After draining excess reagent, the membrane was exposed to X-ray film (Kodak) for 1 h. The film was then developed and the image digitized with appropriate equipment. Two membranes were prepared for experiment replication. For one, cDNA obtained from cells grown in culture medium was hybridized first, followed by the cDNA obtained from cells grown under *in planta *conditions. In the other membrane, the opposite order of hybridization was performed: cDNA obtained from cells grown under *in planta *conditions was hybridized first, followed by cDNA obtained from cells grown in culture medium. In both situations, the probe was removed from the membrane using boiling 0.1% SDS, and the membrane was kept in this solution during cooling to room temperature.

## Authors' contributions

MLL, JD and JBB carried out *in vitro *mutagenesis, mutant library construction and *in vivo *virulence test. MLL and CBF carried out growth curves. MLL and JBB carried out Southern blotting experiments. MLL was responsible for customizing a protocol for and extracting the total DNA, identification of mutated genes, nucleic acid hybridization using labeled cDNA probes and general coordination of the study. MITF and JCFO coordinated and oversaw the project. JAF and ACRS conceived the project. MLL, LMM and JAF were responsible for most data interpretation and final manuscript elaboration. All authors read and approved the final manuscript.

## Supplementary Material

Additional file 1 **Characterization of XCC mutants^a^.** This table provides symptoms, ORF's identification code, gene's name, mutant's identification code, transposon insertion site, and functional category for the 44 mutants. Additionally, mutants with growth curves and gene expression are indicated. Click here for file

## References

[B1] Schaad NW Postnikova E Lacy G Sechler A Agarkova I Stromberg PE Stromberg VK Vidaver AK Emended classification of xanthomonad pathogens on citrus Systematic and Applied Microbiology 2006 29 8 690 695 10.1016/j.syapm.2006.08.001 17183629

[B2] Whiteside J Garnsey S Timmer L Compendium of citrus diseases 1988 Saint Paul: APS Press

[B3] Feichtenberger E Donadio L Manejo ecológico das principais doenças fúngicas e bacterianas dos citros no Brasil Anais do V Seminário Internacional de Citros – Tratos Culturais 1998 Bebedouro: Fundação Cargill 517

[B4] da Silva ACR Ferro JA Reinach FC Farah CS Furlan LR Quaggio RB Monteiro-Vitorello CB Sluys MAV Almeida NF Alves LMC do Amaral AM Bertolini MC Camargo LEA Camarotte G Cannavan F Cardozo J Chambergo F Ciapina LP Cicarelli RMB Coutinho LL Cursino-Santos JR El-Dorry H Faria JB Ferreira AJS Ferreira RCC Ferro MIT Formighieri EF Franco MC Greggio CC Gruber A Katsuyama AM Kishi LT Leite RP Lemos EGM Lemos MVF Locali EC Machado MA Madeira AMBN Martinez-Rossi NM Martins EC Meidanis J Menck CFM Miyaki CY Moon DH Moreira LM Novo MTM Okura VK Oliveira MC Oliveira VR Pereira HA Rossi A Sena JAD Silva C de Souza RF Spinola LAF Takita MA Tamura RE Teixeira EC Tezza RID dos Santos MT Truffi D Tsai SM White FF Setubal JC Kitajima JP Comparison of the genomes of two *Xanthomonas *pathogens with differing host specificities Nature 2002 417 6887 459 463 10.1038/417459a 12024217

[B5] Goryshin IY Jendrisak J Hoffman LM Meis R Reznikoff WS Insertional transposon mutagenesis by electroporation of released Tn5 transposition complexes Nature Biotechnology 2000 18 97 100 10.1038/72017 10625401

[B6] Schmidt H Hensel M Pathogenicity islands in bacterial pathogenesis Clinical Microbiology Review 2004 17 14 56 10.1128/CMR.17.1.14-56.2004 PMC321463 14726454

[B7] Krysan PJ Young JC Sussman MR T-DNA as an insertional mutagen in *Arabidopsis* Plant Cell 1999 11 12 2283 2290 10590158 10.1105/tpc.11.12.2283 PMC144136

[B8] Brown JS Holden DW Insertional mutagenesis of pathogenic fungi Current Opinion in Microbiology 1998 1 4 390 394(5) 10.1016/S1369-5274(98)80054-4 10066509

[B9] de Jesus Ferreira MC Bao X Laizé V Hohmann S Transposon mutagenesis reveals novel loci affecting tolerance to salt stress and growth at low temperature Current Genetics 2001 40 27 39 10.1007/s002940100237 11570514

[B10] Hudson P Gorton TS Papazisi L Cecchini K Frasca S Geary SJ Identification of a virulence-associated determinant, dihydrolipoamide dehydrogenase (lpd), in *Mycoplasma gallisepticum *through in vivo screening of transposon mutants Infection and Immunity 2006 74 2 931 939 10.1128/IAI.74.2.931-939.2006 PMC1360363 16428737

[B11] Laasik E Ojarand M Pajunen M Savilahti H Mäe A Novel mutants of *Erwinia carotovora *subsp. *carotovora *defective in the production of plant cell wall degrading enzymes generated by Mu transpososome-mediated insertion mutagenesis FEMS Microbiology Letters 2005 243 93 99 10.1016/j.femsle.2004.11.045 15668006

[B12] Swarup S De Feyter R Brlansky RH Gabriel DW A pathogenicity locus from *Xanthomonas citri *enables strains from several pathovars of *X. campestris *to elicit cankerlike lesions on citrus Phytopathology 1991 802 809 10.1094/Phyto-81-802

[B13] Yang Y Gabriel DW Intragenic recombination of a single plant pathogen gene provides a mechanism for the evolution of new host specificities Journal of Bacteriology 1995 177 17 4963 8 7665472 10.1128/jb.177.17.4963-4968.1995 PMC177271

[B14] Cornelis GR Van Gijsegem F Assembly and function of type III secretory systems Annual Review of Microbiology 2000 54 735 774 10.1146/annurev.micro.54.1.735 11018143

[B15] Jin Q He SY Role of the Hrp *pilus *in type III protein secretion in *Pseudomonas syringae* Science 2001 294 2556 2558 10.1126/science.1066397 11752577

[B16] Staskawicz BJ Mudgett MB Dangl JL Galan JE Common and contrasting themes of plant and animal diseases Science 2001 292 5525 2285 2289 10.1126/science.1062013 11423652

[B17] Bonas U Schulte R Fenselau S Minsavage GV Staskawicz BJ Isolation of a gene cluster from *Xanthomonas campestris *pv. *vesicatoria *that determines pathogenicity and the hypersensitive response on pepper and tomato Molecular Plant-Microbe Interactions 1991 4 81 88

[B18] Wengelnik K Bonas U *HrpXv*, an AraC-type regulator, activates expression of five of the six loci in the *hrp *cluster of *Xanthomonas campestris *pv. *vesicatoria* Journal of Bacteriology 1996 178 12 3462 3469 8655542 10.1128/jb.178.12.3462-3469.1996 PMC178114

[B19] Wengelnik K Ackerveken G Van den Bonas U HrpG, a key hrp regulatory protein of *Xanthomonas campestris *pv. *vesicatoria *is homologous to two-component response regulators Molecular Plant-Microbe Interactions 1996 9 704 712 8870269 10.1094/mpmi-9-0704

[B20] Rossier O Ackerveken G van den Bonas U HrpB2 and HrpF from *Xanthomonas *are type III-secreted proteins and essential for pathogenicity and recognition by the host plant Molecular Microbiology 2000 38 4 828 838 10.1046/j.1365-2958.2000.02173.x 11115117

[B21] Kim DY Kim KK Structure and function of HtrA family proteins, the key players in protein quality control Journal of Biochemistry and Molecular Biology 2005 38 3 266 274 15943900 10.5483/bmbrep.2005.38.3.266

[B22] Clausen T Southan C Ehrmann M The HtrA family of proteases: implications for protein composition and cell fate Molecular Cell 2002 10 3 443 455 10.1016/S1097-2765(02)00658-5 12408815

[B23] Sassoon N Arie JP Betton JM PDZ domains determine the native oligomeric structure of the DegP (HtrA) protease Molecular Microbiology 1999 33 583 589 10.1046/j.1365-2958.1999.01505.x 10417648

[B24] Wilson RL Brown LL Kirkwood-Watts D Warren TK Lund SA King DS Jones KF Hruby DE *Listeria monocytogenes *10403S HtrA is necessary for resistance to cellular stress and virulence Infection and Immunity 2006 74 765 768 10.1128/IAI.74.1.765-768.2006 PMC1346606 16369036

[B25] Otto M Quorum-sensing control in *Staphylococci *– a target for antimicrobial drug therapy? FEMS Microbiology Letters 2004 241 135 141 10.1016/j.femsle.2004.11.016 15598524

[B26] Cegelski L Marshall GR Eldridge GR Hultgren SJ The biology and future prospects of antivirulence therapies Nature Reviews Microbiology 2008 6 17 27 18079741 10.1038/nrmicro1818 PMC2211378

[B27] Escaich S Antivirulence as a new antibacterial approach for chemotherapy Current Opinion in Chemical Biology 2008 12 4 400 408 10.1016/j.cbpa.2008.06.022 18639647

[B28] Hamza I Chauhan S Hassett R O'Brian MR The bacterial IRR protein is required for coordination of heme biosynthesis with iron availability Journal of Biological Chemistry 1998 273 34 21669 21674 10.1074/jbc.273.34.21669 9705301

[B29] Gilles-Gonzalez MA Ditta GS Helinski DR A haemoprotein with kinase activity encoded by the oxygen sensor of *Rhizobium meliloti* Nature 1991 350 6314 170 172 10.1038/350170a0 1848683

[B30] Verma A Hirsch DJ Glatt CE Ronnett GV Snyder SH Carbon monoxide: a putative neural messenger Science 1993 259 5093 381 384 10.1126/science.7678352 7678352

[B31] Lathrop JT Timko MP Regulation by heme of mitochondrial protein transport through a conserved amino acid motif Science 1993 259 5094 522 525 10.1126/science.8424176 8424176

[B32] Beale SI Neidhardt FC, III RC, Ingraham JL, Lin ECC, Low KB, Magasanik B, Reznikoff WS, Riley M, Schaechter M, Umbarger HE Biosynthesis of Hemes Escherichia coli and Salmonella: Cellular and Molecular Biology 1996 2 Washington, DC: ASM Press 731 748

[B33] Kajie SI Anraku Y Purification of a hexaheme cytochrome c_552 _from *Escherichia coli *K12 and its properties as a nitrite reductase European Journal of Biochemistry 1986 154 457 463 10.1111/j.1432-1033.1986.tb09419.x 3002798

[B34] Kohler C von Eiff C Peters G Proctor RA Hecker M Engelmann S Physiological characterization of a heme-deficient mutant of *Staphylococcus aureus *by a proteomic approach Journal of Bacteriology 2003 185 6928 6937 14617657 10.1128/JB.185.23.6928-6937.2003 PMC262702

[B35] Qian W Han ZJ He C Two-component signal transduction systems of *Xanthomonas *spp.: a lesson from genomics Molecular Plant-Microbe Interactions 2008 21 2 151 161 10.1094/MPMI-21-2-0151 18184059

[B36] Mascher T Helmann JD Unden G Stimulus perception in bacterial signal-transducing histidine kinases Microbiology and Molecular Biology Reviews 2006 70 4 910 938 10.1128/MMBR.00020-06 PMC1698512 17158704

[B37] Dow M Diversification of the function of cell-to-cell signaling in regulation of virulence within plant pathogenic xanthomonads Science Signaling 2008 1 21 pe23 10.1126/stke.121pe23 18506032

[B38] Finn RD Tate J Mistry J Coggill PC Sammut SJ Hotz HR Ceric G Forslund K Eddy SR Sonnhammer ELL Bateman A The Pfam protein families database Nucleic Acids Research 2008 36 Database D281 D288 18039703 10.1093/nar/gkm960 PMC2238907

[B39] Nakai K Horton P PSORT: a program for detecting sorting signals in proteins and predicting their subcellular localization Trends in Biochemical Sciences 1999 24 34 36 10.1016/S0968-0004(98)01336-X 10087920

[B40] Altschul SF Madden TL Schaffer AA Zhang JH Zhang Z Miller W Lipman DJ Gapped BLAST and PSI-BLAST: a new generation of protein database search programs Nucleic Acids Research 1997 25 17 3389 3402 9254694 10.1093/nar/25.17.3389 PMC146917

[B41] He YW Zhang LH Quorum sensing and virulence regulation in *Xanthomonas campestris* FEMS Microbiology Reviews 2008 32 5 842 857 10.1111/j.1574-6976.2008.00120.x 18557946

[B42] Slater H Alvarez-Morales A Barber CE Daniels MJ Dow JM A two-component system involving an HD-GYP domain protein links cell-cell signalling to pathogenicity gene expression in *Xanthomonas campestris* Molecular Microbiology 2000 38 5 986 1003 10.1046/j.1365-2958.2000.02196.x 11123673

[B43] Wang LH He Y Gao Y Wu JE Dong YH He C Wang SX Weng LX Xu JL Tay L Fang RX Zhang LH A bacterial cell-cell communication signal with cross-kingdom structural analogues Molecular Microbiology 2004 51 3 903 912 10.1046/j.1365-2958.2003.03883.x 14731288

[B44] Barber CE Tang JL Feng JX Pan MQ Wilson TJ Slater H Dow JM Williams P Daniels MJ A novel regulatory system required for pathogenicity of *Xanthomonas campestris *is mediated by a small diffusible signal molecule Molecular Microbiology 1997 24 3 555 566 10.1046/j.1365-2958.1997.3721736.x 9179849

[B45] He YW Xu M Lin K Ng YJA Wen CM Wang LH Liu ZD Zhang HB Dong YH Dow JM Zhang LH Genome scale analysis of diffusible signal factor regulon in *Xanthomonas campestris *pv. *campestris*: identification of novel cell-cell communication-dependent genes and functions Molecular Microbiology 2006 59 2 610 622 10.1111/j.1365-2958.2005.04961.x 16390454

[B46] Ryan RP Fouhy Y Lucey JF Crossman LC Spiro S He YW Zhang LH Heeb S Cámara M Williams P Dow JM Cell-cell signaling in *Xanthomonas campestris *involves an HD-GYP domain protein that functions in cyclic di-GMP turnover Proceedings of the National Academy of Sciences of the United States of America 2006 103 17 6712 6717 16611728 10.1073/pnas.0600345103 PMC1458946

[B47] Andrade MO Alegria MC Guzzo CR Docena C Rosa MCP Ramos CHI Farah CS The HD-GYP domain of RpfG mediates a direct linkage between the Rpf quorum-sensing pathway and a subset of diguanylate cyclase proteins in the phytopathogen *Xanthomonas axonopodis *pv. *citri* Molecular Microbiology 2006 62 2 537 551 10.1111/j.1365-2958.2006.05386.x 17020586

[B48] Koonin EV Makarova KS Aravind L Horizontal gene transfer in prokaryotes: quantification and classification Annual Review of Microbiology 2001 55 709 742 10.1146/annurev.micro.55.1.709 11544372 PMC4781227

[B49] Lima WC Sluys MAV Menck CFM Non-gamma-proteobacteria gene islands contribute to the *Xanthomonas *genome OMICS 2005 9 2 160 172 10.1089/omi.2005.9.160 15969648

[B50] Moreira LM Souza RFD Digiampietri LA da Silva ACR Setubal JC Comparative analyses of *Xanthomonas *and *Xylella *complete genomes OMICS 2005 9 43 76 10.1089/omi.2005.9.43 15805778

[B51] Alegria MC Souza DP Andrade MO Docena C Khater L Ramos CHI da Silva Ana CR Farah CS Identification of new protein-protein interactions involving the products of the chromosome- and plasmid-encoded type IV secretion loci of the phytopathogen *Xanthomonas axonopodis *pv. *citri* Journal of Bacteriology 2005 187 2315 2325 15774874 10.1128/JB.187.7.2315-2325.2005 PMC1065226

[B52] Tatusov RL Fedorova ND Jackson JD Jacobs AR Kiryutin B Koonin EV Krylov DM Mazumder R Mekhedov SL Nikolskaya AN Rao BS Smirnov S Sverdlov AV Vasudevan S Wolf YI Yin JJ Natale DA The COG database: an updated version includes eukaryotes BMC Bioinformatics 2003 4 41 12969510 10.1186/1471-2105-4-41 PMC222959

[B53] Sambrook J Fritsch EF Maniatis T Molecular Cloning – A Laboratory Manual 1989 2 New York: Cold Spring Harbor Laboratory Press

[B54] Romeiro RS Bactérias Fitopatogênicas 2005 2 Viçosa: Editora UFV

