# Supplementary material for: New genes of Xanthomonas citri subsp. citri involved in pathogenesis and adaptation revealed by a transposon-based mutant library
Source: BMC Microbiol. 2009 Jan 16;9:12. doi: 10.1186/1471-2180-9-12 (PMC2653504; doi:10.1186/1471-2180-9-12)
Supplement: Additional file 1 — Characterization of XCC mutantsa. This table provides symptoms, ORF's identification code, gene's name, mutant's identification code, transposon insertion site, and functional category for the 44 mutants. Additionally, mutants with growth curves and gene expression are indicated. [file 1471-2180-9-12-S1.pdf]

Table 2 - Characterization of XCC mutants[a]

PHENOTYPE CARACTERIZATION	XCC ID (Psort/SignalP)[b] PRODUCT (gene name) MUTANT(s) DOMAIN STRUCTURE[c]	PHENOTYPE				NB[d]	R[e]	Cf[f]
		WS	HYP	NEC	HR-l			
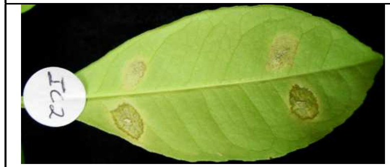	**XAC0014 (IM/NS)** Cardiolipin synthetase (cls) **19C02 / IC02** 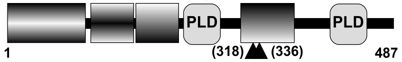	ND	ND	ND	+	—	X	II.D.12
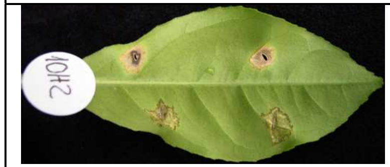	**XAC0095 (BC/NS)** Xanthomonas conserved hypothetical protein **10H02*** 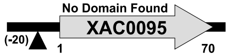	0	-	+	ND	X	X	VIII.C
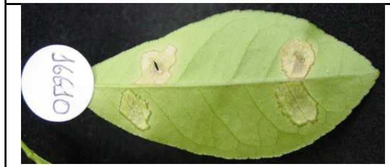	**XAC0102 (BC/NS)** Conserved hypothetical protein **16G10** 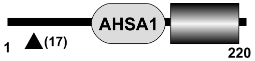	0	-	+	ND	X	—	VIII.A
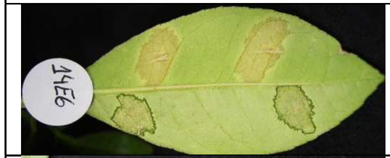	**XAC0144 (OU+PS/SP)** TonB-dependent receptor (iroN) **14E06** 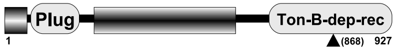	0	-	+	ND	—	—	V.A.7
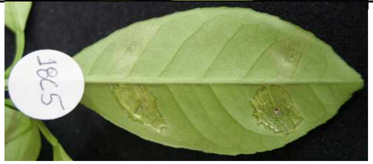	**XAC0340 (BC/NS)** Conserved hypothetical protein **18C05** 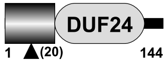	0	0	0	0	X	X	VIII.A

Continue in the next page

Table 2 – continued

PHENOTYPE CARACTERIZATION	XCC ID (Psort/SignalP)[b] PRODUCT (gene name) MUTANT(s) DOMAIN STRUCTURE[c]	PHENOTYPE				NB[d]	R[e]	Cf[f]
		WS	HYP	NEC	HR-l			
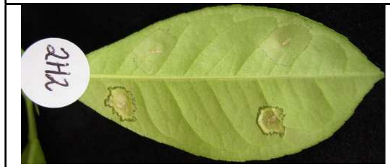	**XAC0410 (IM/NS)**<br>HrpB4 protein (hrpB4)<br>**02H02**<br>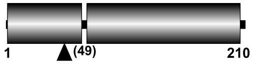	0	0	0	0	—	X	VII.B
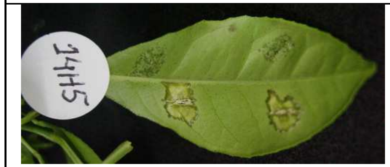	**XAC0618 (IM/NS)**<br>Periplasmic glucan biosynthesis protein (hrpM)<br>**14H05**<br>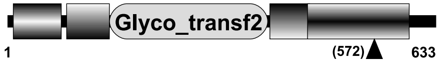	-	-	0	ND	—	—	VII.G
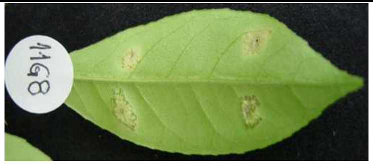	**XAC0756 (IM/SP)**<br>Potassium-transporting ATPase A chain (kdpA)<br>**11G08**<br>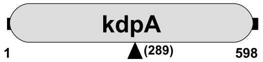	ND	-	+	ND	—	—	V.A.7
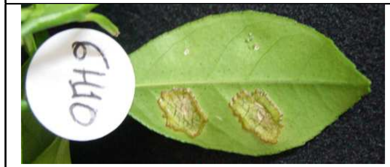	**XAC0789 (BC/NS)**<br>DGTP-pyrophosphohydrolase Thiamine phosphate synthase<br>**06H10**<br>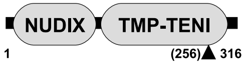	0	0	0	0	—	X	III.A.4
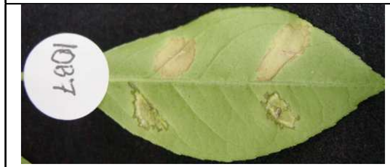	**XAC0798 (OM+PS/SP)**<br>Alpha-amylase (amy)<br>**10B07**<br>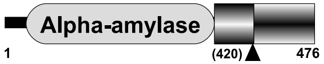	ND	ND	ND	+	—	—	I.A.1

Continue in the next page

Table 2 – continued

PHENOTYPE CARACTERIZATION	XCC ID (Psort/SignalP)[b] PRODUCT (gene name) MUTANT(s) DOMAIN STRUCTURE[c]	PHENOTYPE				NB[d]	R[e]	Cf[f]
		WS	HYP	NEC	HR-l			
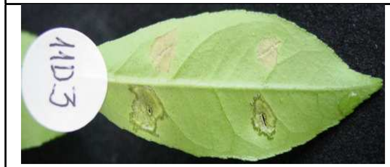	**XAC1201 (IM/NS)**<br>Conserved hypothetical protein<br>**11C09 / 11D03**<br>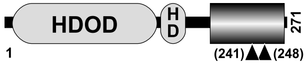	ND	ND	ND	+	—	X	VIII.A
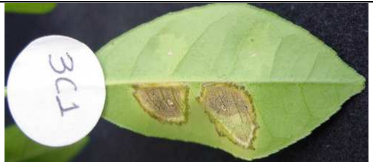	**XAC1266 (BC/NS)**<br>HrpX protein (hrpXct)<br>**03C01**<br>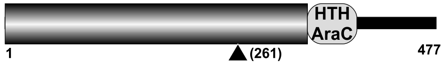	0	0	0	0	—	X	VII.B
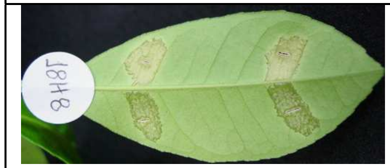	**XAC1669 (BC/NS)**<br>Histidine kinase-response regulator hybrid protein<br>**18H08**<br>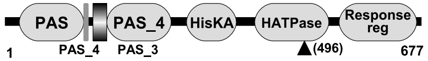	-	-	+	ND	—	—	I.D.3
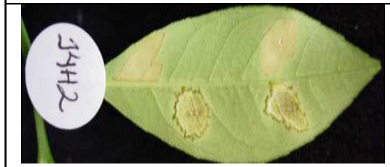	**XAC1927 (IM/NS)**<br>Fe-S oxidoreductase (aslB)<br>**14H02 / 25D11**<br>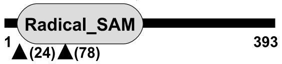	ND	ND	ND	+	X	—	I.C.3
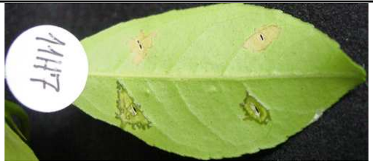	**XAC2047 (BC/NS)**<br>PHA synthase subunit<br>**10H09 / 11A04 / 11H07**<br>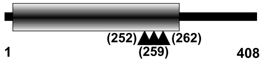	ND	ND	ND	+	X	—	IX

Continue in the next page

Table 2 – continued

PHENOTYPE CARACTERIZATION	XCC ID (Psort/SignalP)[b]<br>PRODUCT (gene name)<br>MUTANT(s)<br>DOMAIN STRUCTURE[c]	PHENOTYPE				NB[d]	R[e]	Cf[f]
		WS	HYP	NEC	HR-l			
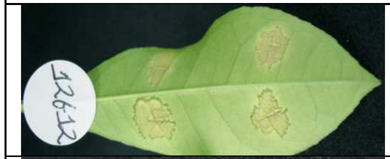	**XAC2053 (IM/NS)**<br>Transcription-related protein (tex)<br>**12G12**<br>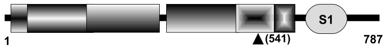	ND	ND	ND	+	X	—	I.D.2
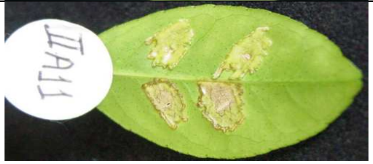	**XAC2072 (BC/NS)**<br>Sugar ABC transporter<br>ATP-binding protein (ugpC)<br>**18H02 / 18H11 / IIA11**<br>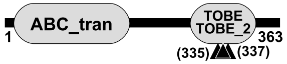	++	++	0	ND	—	—	V.A.3
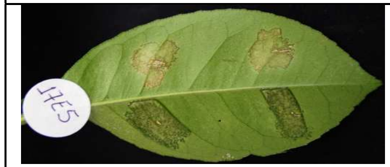	**XAC2118 (IM/NS)**<br>Conserved hypothetical protein<br>**17E05**<br>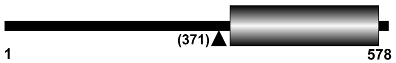	-	-	+	ND	—	—	VIII.A
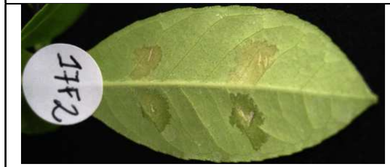	**XAC2639 (BC/NS)**<br>Site-specific DNA methyltransferase<br>**17F02**<br>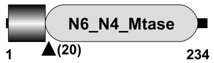	0	-	+	ND	—	—	II.A.5
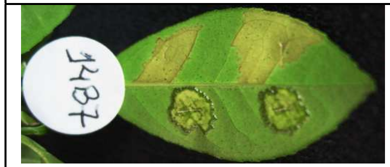	**XAC3136 (BC/NS)**<br>Two-component system<br>sensor protein (exsG)<br>**14B07***<br>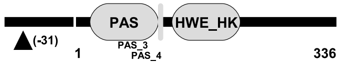	ND	ND	ND	+	—	—	I.D.1

Continue in the next page

Table 2 – continued

PHENOTYPE CARACTERIZATION	XCC ID (Psort/SignalP)[b] PRODUCT (gene name) MUTANT(s) DOMAIN STRUCTURE[c]	PHENOTYPE				NB[d]	R[e]	Cf[f]
		WS	HYP	NEC	HR-l			
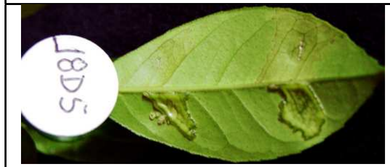	**XAC3225 (PS+OM/SP)** Transglycosylase (mltB) **18D05** 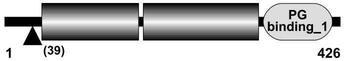	-	-	-	ND	X	—	IV.A.2
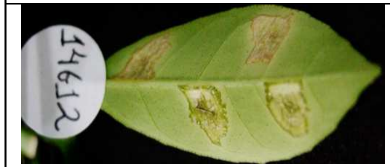	**XAC3245 (OM+PS/NS)** RhsD protein **14G01 / 14G12** 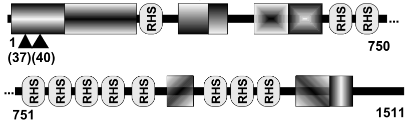	ND	ND	+	ND	—	—	IX
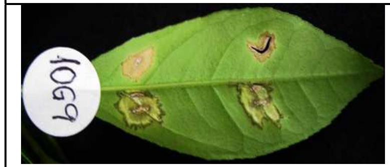	**XAC3263 (BC/NS)** Hypothetical protein **10G07 / 10G09** 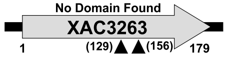	0	-	+	ND	X	—	VIII.B
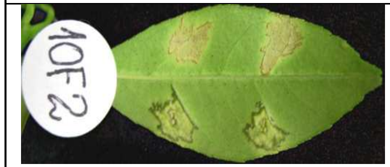	**XAC3285 (BC/NS)** Hypothetical protein **10F02** 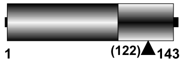	0	-	+	ND	X	—	VIII.B
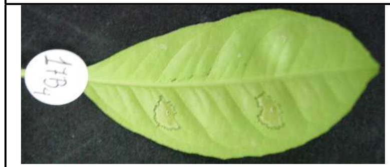	**XAC3294 (BC/NS)** Hypothetical protein **17B04** 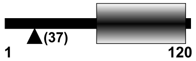	0	0	0	0	—	—	VIII.B

Continue in the next page

Table 2 – continued

PHENOTYPE CARACTERIZATION	XCC ID (Psort/SignalP)[b] PRODUCT (gene name) MUTANT(s) DOMAIN STRUCTURE[c]	PHENOTYPE				NB[d]	R[e]	Cf[f]
		WS	HYP	NEC	HR-l			
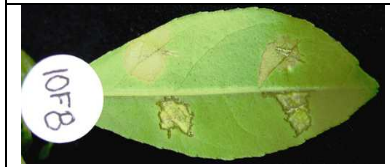	**XAC3320 (BC/NS)**<br>ISxac3 transposase<br>**10F08**<br>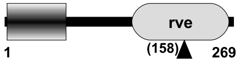	ND	ND	ND	+	—	—	VI.C
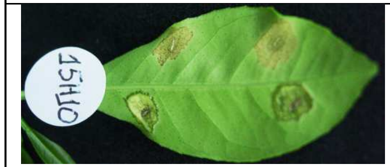	**XAC3457 (BC/NS)**<br>3-isopropylmalate dehydratase subunit (leuD)<br>**15H10**<br>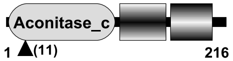	ND	ND	ND	+	X	—	II.A.2
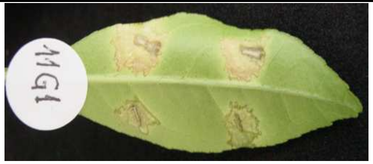	**XAC3581 (BC/NS)**<br>UDP-glucose dehydrogenase (ugd)<br>**11G01**<br>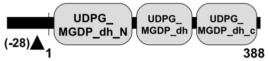	0	-	+	ND	–	—	I.B.11
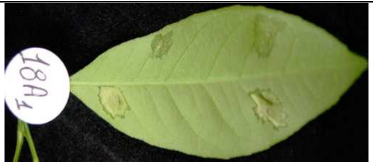	**XAC3600 (BC/NS)**<br>ABC transporter ATP-binding protein (wzt)<br>**18A01**<br>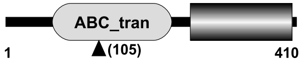	++	++	0	ND	—	—	V.A.7
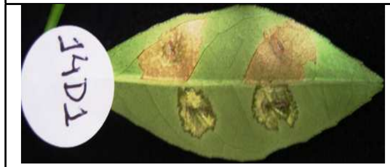	**XAC3607 (BC/NS)**<br>Type II secretion system protein-like protein (uptC)<br>**14D01**<br>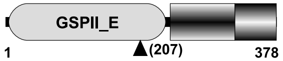	ND	ND	ND	+	—	—	IV.A.2

Continue in the next page

Table 2 – continued

PHENOTYPE CARACTERIZATION	XCC ID (Psort/SignalP)[b]<br>PRODUCT (gene name)<br>MUTANT(s)<br>DOMAIN STRUCTURE[c]	PHENOTYPE				NB[d]	R[e]	Cf[f]
		WS	HYP	NEC	HR-l			
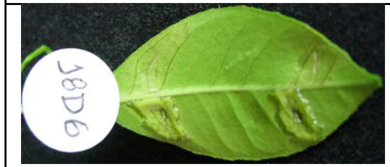	**XAC3673 (IM/NS)**<br>Histidine kinase<br>**18D06**<br>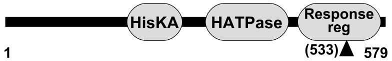	0	0	0	0	—	X	I.D.3
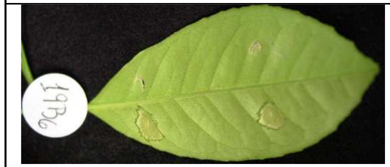	**XAC3980 (OM+PS/SP)**<br>Protease DO (htrA)<br>**19B06**<br>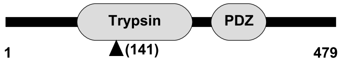	0	0	0	0	—	—	III.C.3
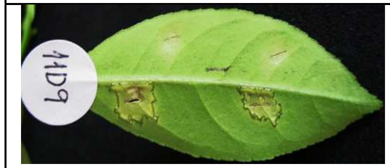	**XAC4040 (BC/NS)**<br>Delta-aminolevulinic acid dehydratase (hemB)<br>**11D09**<br>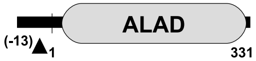	0	0	0	0	—	X	II.D.12
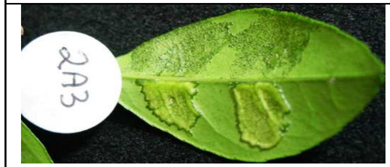	**XAC4160 (IM/SP)**<br>Cation efflux system protein (czcA)<br>**02A03**<br>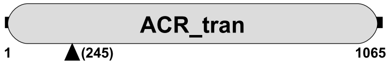	-	-	0	ND	—	—	VIII.C
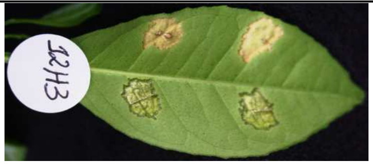	**XACb0067 (IM/NS)**<br>Tn5045 transposase<br>**12H03**<br>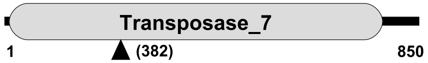	ND	-	+	ND	—	—	VI.C

[a] Data obtained from *Xanthomonas citri* subsp. *citri* genome web page; [b] Psort (http://psort.nibb.ac.jp/form.html) and SignalP (http://www.cbs.dtu.dk/services/SignalP/) identification (IN – inner membrane, OM – outer membrane, BC – bacterial cytoplasm, PS – periplasmic space, NS – non-secretory, SP – signal peptide); [c] Pfam domain architecture (names listed below); [d] northern blotting analysis

and [e] reactivation curve in plant and in medium culture (“X” - available; “—” - not available); [f] Functional categorization based on genome annotation (http://genoma4.fcav.unesp.br/xanthomonas/). The black triangles identify the site of transposition (EZ::Tn KAN-2) for each mutant, and the number represents the specific site of mutation; PHENOTYPE: WS - water soaking formation; Hyp - hyperplasia, Nec - necrosis, HR-l - hypersensitive-like response (“ND” - not defined; “+” - induction; “++” - overinduction; “-” - repression; “0” - total absence of symptoms). Domains: PLD - phospholipase D active site motif (PF006140); AHAS1 - activator of Hsp90 ATPase homolog 1-like protein (PF08327); Plug - TonB-dependent receptor plug domain (PF07715); TonB_dep_rec - TonB dependent receptor (PF00593); DUF24 - HxlR-like helix-turn-helix (PF01638); Glyco_transfer2 - glycosyl transferase family 2 (PF00535); kdpA - potassium-transporting ATPase A subunit (PF03814); NUDIX - NUDIX domain (PF00293); TMP-TENIdependent receptor plug domain (PF07715) thiamine monophosphate synthase/TENI (PF02581); Alpha_amylase - alpha-amylase, catalytic domain (PF00128); HDOD - HDOD domain (PF08668); HD - HD domain (PF01966); HTH-AraC - bacterial regulatory helix-turn-helix proteins, AraC family (PF00165); PAS - PAS fold (PF00989); PAS_3 - PAS fold (PF08447); PAS_4 - PAS fold (PF08448); HisKA - histidine kinase A (phosphoacceptor) domain (PF00512) ; HATPase - histidine kinase-, DNA gyrase B-, and HSP90-like ATPase (PF02518); Response_reg - response regulator receiver domain (PF00072); Radical_SAM - radical SAM superfamily (PF04055); S1 - S1 RNA binding domain (PF00575); ABC_tran - ABC transporter (PF00005); TOBE - TOBE domain (PF03459); TOBE_2 - TOBE domain (PF08402); N6_N4_Mtase - DNA methylase (PF01555); HWE_HK - HWE histidine kinase (PF07536); PG_binding_1 - putative peptidoglycan binding domain (PF01471); RHS - RHS repeat (PF05593); rve - integrase core domain (PF00665); Aconitase_c - aconitase C-terminal domain (PF00694); UDPG-MGDP-dh_N - UDP-glucose/GDP-mannose dehydrogenase family, NAD binding domain (PF03721); UDPG-MGDP-dh - UDP-glucose/GDP-mannose dehydrogenase family, central domain (PF00984); UDPG-MGDP-dh_C - UDP-glucose/GDP-mannose dehydrogenase family, UDP binding domain(PF03720); GSPII_E - Type II/IV secretion system protein (PF00437); UDG - uracil DNA glycosylase superfamily (PF03167); Trypsin - trypsin (PF00089); PDZ - PDZ domain, also known as DHR or GLGF (PF00595); ALAD - delta-aminolevulinic acid dehydratase (PF00490); ACR_tran - AcrB/AcrD/AcrF family (PF00873); Transposase_7 - transposase (PF01526).
